# Cognitive behavioural therapy compared to control conditions in treating bulimia nervosa and binge‐eating disorder: A systematic review and meta‐analysis

**DOI:** 10.1111/bjc.70070

**Published:** 2026-05-22

**Authors:** Angeliki L. Sioziou, Anastasios Stefanou, Felicia Dimitrakopoulou, Menelaos Skarlatos, Andreas S. Lappas, Christina Mesiari, Aikaterini Argyrou, Evangelos Ntouros, Nikolaos Christodoulou, Vasilios‐Panteleimon Bozikas, Myrto Samara

**Affiliations:** ^1^ Department of Psychiatry, Faculty of Medicine University of Thessaly Larisa Greece; ^2^ Second Department of Psychiatry, Faculty of Health Sciences, School of Medicine Aristotle University of Thessaloniki Thessaloniki Greece; ^3^ Department of General Psychology University of Padua Padova Italy; ^4^ Department of Geriatric Psychiatry Aneurin Bevan University Health Board Newport UK

**Keywords:** binge eating episodes, eating disorders, psychotherapy, purging, treatment

## Abstract

**Objective:**

Our systematic review and meta‐analysis aimed to investigate how Cognitive‐Behavioural Therapy (CBT) performs against control conditions, such as waiting‐list, self‐help interventions, psychological and pill placebos and treatment as usual (TAU) in Bulimia Nervosa (BN) and Binge Eating Disorder (BED).

**Methods:**

We systematically searched MEDLINE, Embase, PsycINFO, Cochrane CENTRAL, WHO ICTRP, ClinicalTrials.gov for randomized controlled trials (RCTs) comparing CBT to any control condition, in patients with BED and BN. Analyses employed random‐effects models, calculating risk ratios (RR) for dichotomous outcomes, weighted mean differences (MD) for continuous outcomes and standardized mean differences (SMD) when scales varied. The primary outcomes were remission and binge eating frequency. Subgroup and sensitivity analyses assessed robustness.

**Results:**

42 RCTs (2807 participants randomized) were included. CBT demonstrated superiority over waitlists in achieving remission (RR = 3.21, 95% CI: 2.15; 4.81) and in reducing binge‐eating frequency (SMD = −.88, 95% CI: −1.08; −.69). However, compared to self‐help, effects were attenuated for remission (RR = 1.53, 95% CI 1.19; 1.95) and were not significant for binge frequency (SMD = −.14, 95% CI: −.32; .04). Data were scarce for other inactive comparators.

**Discussion:**

The superiority of CBT is contingent on the comparator used, highlighting the necessity for rigorous controls such as a psychological placebo or other therapy modalities.

**Practitioner points**

- CBT presents robust efficacy over waitlist controls, but these results are compromised when CBT is compared to self‐help interventions.
- Evidence remains limited for comparisons with treatment‐as‐usual, psychological placebos and other active comparators.
- Future research should employ head‐to‐head trials comparing CBT with active controls and/or other structured therapies, in order to isolate its specific therapeutic effects.

## INTRODUCTION

Cognitive Behavioural Therapy (CBT) is the first‐line treatment for Bulimia nervosa (BN) and Binge Eating Disorder (BED) as suggested by all treatment guidelines (Crone et al., 2023; Hay et al., 2014; National Institute of Health and Care Excellence, 2017). This aligns with the fact that preoccupation with body size and shape, a core characteristic of both disorders, constitutes a cognitive distortion (Murphy et al., 2010). Evidence suggests that this intervention leads to remission in around 50% of patients with these disorders (Agras, 2019; Wilson et al., 2007).

Usually, 20 sessions are conducted following a 3‐stage protocol, where the patient is first introduced to recognising symptoms and relevant conditions, then guided to a cognitive restructuring of thoughts about body weight and stress management and, finally, educated about managing a possible relapse, as well as maintaining a healthy dietary behaviour (Grilo, 2017; Lilly, 2003).

Existing meta‐analyses comparing psychotherapy to waitlist controls consistently show the superiority of CBT for outcomes such as remission and the reduction of purging or binge‐eating behaviours (Cuijpers et al., 2025). However, evidence from comparisons with other active psychological treatments is less definitive. The number of trials is low, and existing results, while showing no significant difference, are uncertain (Grenon et al., 2017, 2019; Linardon et al., 2018; Polnay et al., 2014; Whittal et al., 1999). Meanwhile, a recent meta‐analysis shows CBT to be as effective as pharmacological interventions (Samara et al., 2024).

To our knowledge, no meta‐analysis has yet evaluated the efficacy of CBT specifically against a full range of control conditions—including self‐help, treatment as usual (TAU), psychological or pill placebo and no‐treatment—in patients with BN or BED. A recent synthesis by Bruns et al. found CBT comparable to a variety of other psychotherapeutic approaches, such as interpersonal therapy, mindfulness‐based or psychoanalytic therapies, though evidence specific to BN and BED was limited (Bruns et al., 2025). However, this analysis did not examine the differential impact of specific control conditions beyond broad categories (e.g., waitlist) or other psychotherapies represented by scarce RCTs. Moreover, CBT was defined broadly to include both therapist‐led and self‐help formats. The distinction between these formats, as well as the role of comparator type, remains of particular relevance when interpreting treatment effects.

Control conditions in RCTs are used to increase internal validity by isolating the specific effects of an intervention from the natural course of the illness, including spontaneous improvement, fluctuation, regression to the mean and observer expectancy effects (Bruun, 2022; Goldberg et al., 2023; Mohr et al., 2009). Nevertheless, in psychotherapy trials, controls such as waiting lists or no‐treatment can inflate effect sizes, possibly due to a nocebo effect (Furukawa et al., 2014; Michopoulos et al., 2021; Patterson et al., 2016).

A placebo is considered the gold standard comparator. But, in psychotherapy trials, a psychological placebo is difficult to define, given the need for a control that may match the intervention on key non‐specific therapeutic factors (Mohr et al., 2009).

The aim of this meta‐analysis is to provide a comprehensive evaluation of the efficacy of CBT relative to key control conditions, including waitlist, self‐help, TAU, psychological placebo, pill placebo and no‐treatment. This direct comparison will help elucidate the specific components of CBT that contribute to treatment efficacy for BN or BED.

## METHOD

This review was conducted according to the Preferred Reporting Items for Systematic Reviews and Meta‐analyses (PRISMA) statement (Table S1) (Moher et al., 2009).

### Protocol

A pre‐designed protocol was used to conduct this systematic review and meta‐analysis, which was also submitted electronically in the Open Science Framework (Project URL: OSF View only link) and is presented in the Supplementary Material (Supplementary Material, Section 1).

### Selection criteria: Population, intervention, comparator, and types of included studies

Individuals diagnosed with BED or BN were included, irrespective of the diagnostic system applied. No restrictions were placed regarding age, gender, possible comorbidities and number of participants. The intervention of interest was therapist‐led CBT; we included all modalities of CBT delivery (e.g., individual, group, tele‐therapy) but excluded interventions based solely on self‐help techniques, books, unguided digital applications or weight loss techniques. The comparator intervention was any of the following: guided or unguided self‐help, TAU, psychological placebo, pill placebo, no treatment or a waiting list for psychotherapy.

Eligible studies were only RCTs (excluding cluster and quasi‐RCTs), with no restrictions on blinding methods, reporting any of the outcomes listed below.

### Outcomes measured

The primary outcomes of the study were (i) remission of disorder‐related symptoms, defined in advance as a 100% symptom reduction for at least 2 weeks or until the criteria for diagnosis, including cognitive elements, were not met (Argyrou et al., 2023; Samara et al., 2023) and (ii) the frequency of binges.

We included the following secondary outcomes: (i) frequency of purges; (ii) response to treatment, defined as a reduction in disorder symptomatology of at least 50%; we also used the authors' definitions or, if multiple definitions were presented, we used the strictest one as we did in our previous projects (Argyrou et al., 2023; Samara et al., 2023); (iii) psychopathology of BN and BED; (iv) depression; (v) anxiety; (vi) self‐esteem; (vii) quality of life, all measured by validated rating scales; (viii) weight (measured in kilograms); (ix) body mass index (BMI); (x) total dropouts due to any reason, as a measure of acceptability; (xi) dropouts due to adverse events; (xii) dropouts due to inefficacy and (xiii) total number of patients with adverse events.

### Search strategy and study selection process

We systematically searched the following four databases up to December 2025: Medline (via Ovid), EMBASE, Cochrane Control Register of Controlled Trials (CENTRAL), ClinicalTrials.gov. In addition to the above, a search was also conducted regarding the reference lists of any previous meta‐analyses and systematic reviews and all the included RCTs. A detailed search string for Medline can be found in the Supplementary Material (Section 3).

Three independent reviewers (ALS, MS, CM) screened all abstracts and subsequently the relevant full texts from the searches performed, as well as additional records. Each record was assessed independently by all reviewers. This process was conducted using Rayyan (Ouzzani et al., 2016). Any conflicts during the selection process were resolved through extensive discussions among reviewers and, when necessary, with the senior author (MTS).

### Data extraction

The data extraction process was conducted using a predesigned form for the purpose of the study. In case of missing data, contact was made with the corresponding authors.

Three reviewers (ALS, MS, CM) extracted data from the included studies. Each reviewer worked on a subset of studies, so that data extraction was performed twice independently for each study. In case of a disagreement, a fourth reviewer resolved it (MTS). Missing Standard Deviations (SDs) were estimated either from standard errors, confidence intervals (CI) and *p*‐values or they were imputed by the mean SDs of other studies (Furukawa et al., 2006).

### Statistical analysis

A meta‐analysis was performed for the quantitative composition of the data with the use of R studio version 4.4.1 (R Core Team, 2024), including the packages “meta” (version 8.2.1.), “metafor” (version 4.8.0) and “ggplot2” (version 4.0.2). The Random‐Effects Model of meta‐analysis was used (Morlino et al., 2007). This model is typically more conservative in assessing statistical significance, though greater weight may be assigned to smaller studies, which may affect the point estimate of the effect size (Borenstein et al., 2009; Dettori et al., 2022). The meta‐analysis was conducted on an intention‐to‐treat (ITT) basis. Imputed data using mixed models for repeated measures, last observation carried forward or other validated methods were considered preferable to completer or observed‐case analyses. To address potential issues arising from incomplete outcome data, a sensitivity analysis was conducted, excluding all studies at high risk of attrition bias (see Supplementary Section 1 for sensitivity criteria).

The risk ratio (RR) was calculated for dichotomous variables and the mean difference (MD) for continuous outcomes. If an outcome was measured with various units across the included studies (or with the use of different scales), the effect size used was Hedge's adjusted g (standardized mean difference – SMD). Effect sizes are presented along with their 95% CI and Prediction Intervals (PI). The primary outcome of remission is also presented with the number needed to treat metric (NNT), an effect size which allows more accurate clinical interpretation, as it presents the effect of CBT against the control event rate (Mendes et al., 2017). Change‐from‐baseline data were preferred because they exhibit less between‐person variability (Deeks et al., 2024). If these were not available, endpoint values were extracted instead.

Heterogeneity was assessed with the chi‐square test and quantified by I^2^ statistics. We explored reasons for heterogeneity and assessed the robustness of our results through pre‐specified subgroup, meta‐regression and sensitivity analyses. These analyses were conducted for the two primary outcomes: remission and the frequency of binge episodes.

Subgroup analyses were performed based on the following pre‐defined criteria: (i) control condition (waiting list, psychological placebo etc.); (ii) diagnosis (BN vs. BED); (iii) treatment history (previously treated vs. treatment‐naïve participants); (iv) comorbidity status (presence vs. absence of comorbid disorders); (v) risk of allegiance bias (i.e., whether the therapy developers were also trial authors); (vi) risk of attrition bias; (vii) risk of detection bias; (viii) psychotherapy duration (≤6 weeks vs. >6 weeks); (ix) format of CBT delivery (i.e. individual or group CBT). Meta‐regression analyses were conducted a priori to examine the influence of (i) baseline symptom severity (as measured both by baseline scores of eating psychopathology or baseline BMI) and (ii) the duration of psychotherapy (in weeks). Sensitivity analyses were also pre‐planned to test the robustness of the findings by (i) excluding non‐double‐blind studies (i.e., open‐label and single‐blind trials); (ii) excluding studies reporting only completer analyses; (iii) excluding studies with an overall high risk of bias; (iv) applying a fixed‐effects model instead of the primary random‐effects model.

### Risk of bias

In terms of assessing risk of bias, two reviewers (ALS, MS, CM) assessed the risk of bias using the Cochrane risk of bias tool (study based) for randomized trials (RoB) (Higgins et al., 2011). The overall risk of bias for each study was classified as ‘high’, ‘moderate’ or ‘low’ based on the assessment of individual risk of bias components according to Furukawa et al. (2016). The formula for calculating the overall risk of bias can be found in the Supplementary Material (Table S4).

### Publication bias

To address any possible publication bias, we searched for grey literature, such as clinical trial registries and major conferences' abstract lists. For the primary outcomes with a minimum of ten studies, funnel plots were generated and evaluated for symmetry, using the ‘trim and fill’ method and Egger's g test (Duval & Tweedie, 2000; Egger et al., 1997; Page et al., 2024).

### Certainty of evidence

We used Cochrane's GRADE approach to assess the certainty of evidence. A summary of findings table was generated for all primary outcomes, as well as for response to treatment, eating psychopathology and purging frequency (Schünemann et al., 2024).

## RESULTS

### Description of included studies

Forty‐two studies with a total of 2807 participants were included in this meta‐analysis (see Figure S1 for details). The age of participants ranged from 12 to 65 years, with a mean age of 33.7 years; 97.03% were female. 15 studies reported the mean baseline BMI, which ranged from 21.19 to 42.59 kg/m^2^ (mean baseline BMI: 34.04 kg/m^2^). Regarding diagnosis, 18 RCTs included patients with BN, 23 with BED, and three with either diagnosis. The diagnostic criteria employed were primarily based on four different versions of the DSM or ICD‐10. Geographically, 15 were conducted in Europe, 23 in the US, three in Canada and one in Australia. The study duration ranged from six to 24 weeks (median: 16 weeks), and publications spanned from 1986 to 2024. Regarding intervention delivery, CBT was administered in a group format in 23 studies and in individual sessions in eight studies. Control conditions across studies included waiting‐list (*n* = 30), self‐help (*n* = 10), treatment as usual (TAU; n = 1), pill placebo (n = 3) and no treatment (n = 1). A detailed description of the included section can be found in Supplement Section 5.

### Risk of bias assessment

11 studies (26.19%) were judged as having overall unclear risk of bias, while 31 studies (73.81%) were judged as having an overall high risk of bias. No study was at an overall low risk of bias, as therapists could not be blinded in psychotherapy trials, leading to a high risk of performance bias. The risk of bias summary plot and assessment per individual study are presented in the Supplementary Material (Figures S2, S3.1 and S3.2, respectively).

### Primary outcomes

#### Remission

Remission data comparing CBT to relevant comparators were reported in 20 RCTs. Overall, CBT was associated with significantly higher remission rates (RR = 1.89, 95% CI: 1.50; 2.39, PI: .84; 4.23, NNT = 4.7, 20 RCTs, *N* = 1617, Figure 1). The effect size was larger when CBT was compared to a waiting list (RR = 3.21, 95% CI: 2.15; 4.81, NNT = 2.9, 11 RCTs, *N* = 606) than to self‐help (RR = 1.53, 95% CI 1.19; 1.95, NNT = 7.0, 9 RCTs, *N* = 837).

**FIGURE 1:**
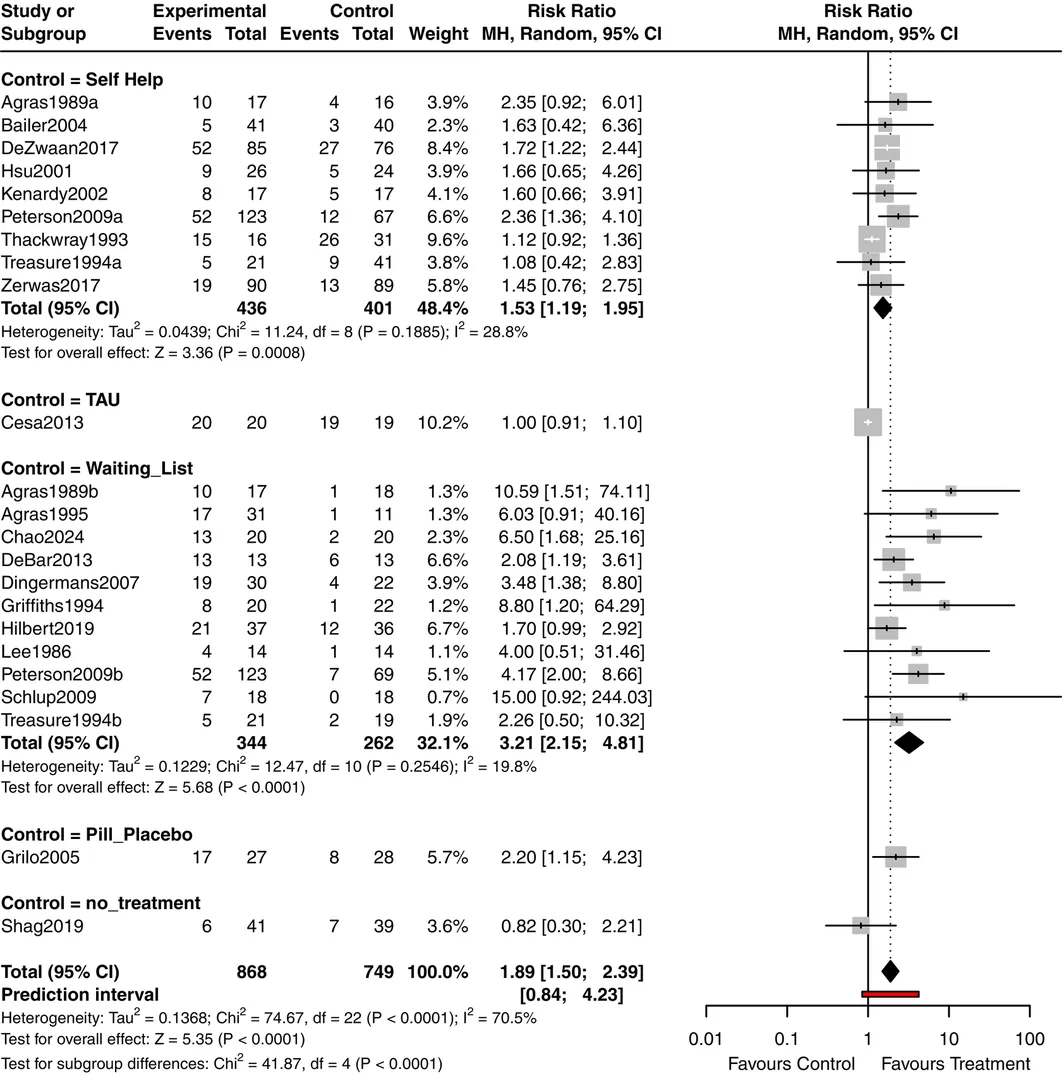
Forest plot—resmission rates, subgroup analysis by control condition. RR, risk ratio with 95% CI (confidence intervals).

#### Frequency of binge‐eating

30 RCTs reported the effect of CBT on the frequency of binging, either measured in episodes or days per week or month. CBT was found to significantly reduce binge behaviours (SMD = −.59, 95% CI: −.77; −.40, PI: −1.51; .33, 30 RCTs, *N* = 2126, Figure 2). Analysis by control condition revealed a large improvement for CBT compared to a waiting list (SMD = −.88, 95% CI: −1.08; −.69, 20 RCTs, *N* = 1096); however, no significant difference was found when CBT was compared to self‐help (SMD = −.14, 95% CI: −.32; .04, 10 RCTs, *N* = 839).

**FIGURE 2:**
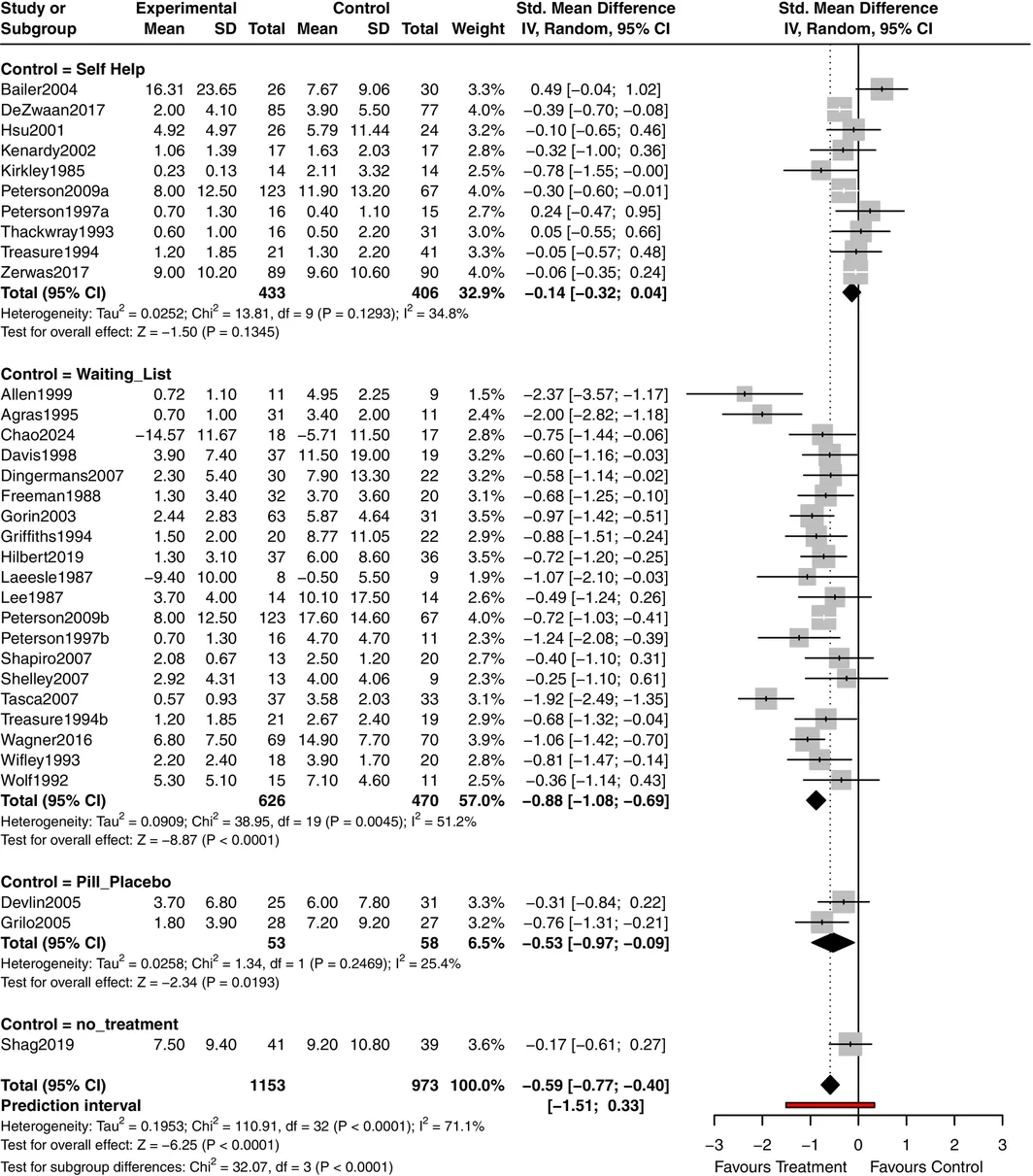
Forest plot—frequency of binge‐eating, subgroup analysis by control condition. SMD, standardized mean difference with 95% CI (confidence intervals).

### Secondary outcomes

#### Response

28 RCTs reported response data comparing CBT with any relevant comparator. CBT was associated with higher response rates (RR = 2.25, 95% CI: 1.67; 3.04, PI: .60; 8.47, 28 RCTs, *N* = 1753, Figure S6).

#### Eating psychopathology

25 RCTs reported the effect of CBT on eating psychopathology, measured by validated scales. CBT was found to improve relevant scale scores, when compared to the included control interventions (SMD = −.39, 95% CI: −.58; −.20, PI: −1.25; .47, 25 RCTs, *N* = 1919, Figure S7).

#### Frequency of purging

Nine RCTs reported on the frequency of purging, either measured in episodes or days per week or month. CBT was found to reduce purging behaviours in comparison to control interventions (SMD = −.55, 95% CI: −.89; −.20, PI: −1.69; .59, 9 RCTs, *N* = 566, Figure S8).

#### Depression

28 studies reported data on depressive symptomatology as measured by validated scales in patients with binge eating disorder or bulimia nervosa. CBT was found to improve symptoms of depression (SMD = −.31, 95% CI: −.49; −.14, PI: −1.13; .50, 28 RCTs, *N* = 2163, Figure S9).

#### Anxiety

Four studies reported data on anxiety symptoms as measured by validated scales in patients with binge eating disorder or bulimia nervosa. CBT was found to marginally reduce anxiety compared to control interventions (SMD = −.17, 95% CI: −.34; −.00, PI: −.44; .10, 4 RCTs, *N* = 558, Figure S10).

#### Self‐esteem

13 studies reported on subjective measurements of self‐esteem with the use of validated scales. CBT was found to have a positive effect on self‐esteem compared to control interventions (SMD = .27, 95% CI: .03; .51, PI: −.56; 1.10, 13 RCTs, *N* = 1188, Figure S11).

#### Quality of life

Six RCTs reported subjective data on quality of life as measured by validated scales. No difference was found on the effect of CBT on this outcome compared to control interventions (SMD = .08, 95% CI: −.04; .21, PI: −.08; .24, 6 RCTs, *N* = 994, Figure S12).

#### Weight

Six studies reported data on weight. No difference was found between CBT and control interventions concerning this outcome (MD = −1.67 kg, 95% CI: −6.34; 3.00, −7.80; 4.45, 6 RCTs, *N* = 331, Figure S13).

#### Body mass index (BMI)

15 studies reported data on BMI. No difference was found between CBT and control interventions concerning this outcome (MD = −.39 kg/m^2^, 95% CI: −1.43; .65, −3.93; 3.15, 15 RCTs, *N* = 1416, Figure S14).

#### Dropouts due to any reason

38 studies reported the number of dropouts per arm as a measure of acceptability. No difference was found between CBT and control interventions concerning this outcome (RR = 1.07, 95% CI: .85; 1.35, PI: .51; 2.24, 38 RCTs, *N* = 2568, Figure S15).

### Subgroup and sensitivity analyses

Results were largely consistent between patients with bulimia nervosa and binge‐eating disorder, and no significant differences emerged across subgroups defined by risk of bias or format of CBT (individual or group therapy). Meta‐regression indicated no significant correlation between primary outcomes and baseline binge frequency or BMI; however, treatment duration was found to moderate the effect of CBT on remission rates (Supplement Section 8). Specifically, longer treatment duration was associated with higher remission rates compared to control interventions. Sensitivity analyses yielded results consistent with the main pooled analyses. Corresponding plots for subgroup and sensitivity analyses are available in the Supplementary Material (Sections 7 and 8).

### Publication bias

Evidence of publication bias for the primary outcome of remission rates was observed, based on visual assessment of funnel plot asymmetry (Figures 2 and S22.1) and a significant Egger's test (*t*‐test = 6.91, *p*‐value <.01, Figure S22.3). The relevant plots can be found in the Supplementary Material (Section 9).

### Certainty of evidence

Based on the assessment of our results with the use of GRADE, a low certainty of evidence was found for all primary outcomes, as well as the outcomes of response to treatment, eating psychopathology and purging frequency. We downgraded our certainty across these outcomes, as most studies had a high risk of bias and a relatively high heterogeneity (Table S5).

## DISCUSSION

To our knowledge, this is the most comprehensive meta‐analysis to date evaluating the efficacy of CBT against various control conditions for binge‐eating spectrum disorders (BSDs). This approach allowed us to investigate how the choice of comparator influences the results.

Pooling data from 42 RCTs (*N* = 2807), we found CBT to be superior to control conditions collectively across most key outcomes, including remission, response, reduction of binge‐eating and purging episodes and improvements in eating‐related psychopathology, depressive symptoms and self‐esteem. Crucially, the evidence base was dominated by comparisons with a waitlist control (30 RCTs). When compared to the 10 RCTs using a self‐help control, CBT's efficacy was markedly attenuated, showing reduced superiority for remission (Pooled NNT of 4.7 increased to 7.0 when compared to self‐help) and no significant advantage for reducing binge frequency (Pooled SMD = −.59, 95% CI: −.77; −.40, to SMD = −.14, 95% CI: −.32; .04 when compared to self‐help). In contrast, CBT remained markedly superior to a waitlist control for both outcomes.

Data for other comparator types were scarce, leaving no room for meaningful clinical interpretation. Specifically, only one RCT used TAU, three used a pill placebo and one used a no‐treatment control. Furthermore, no original study explicitly defined an intervention as a psychological placebo.

Within this methodological context, the finding that the superiority of CBT was less pronounced when compared against self‐help—arguably a more rigorous test of its specific efficacy—is particularly revealing.

The large effect size observed for CBT in the present analysis aligns with the broader literature on these disorders, which has predominantly relied on comparisons with waitlist controls. For instance, studies by Monteleone et al. and Cuijpers et al. have reported similarly substantial effects for CBT against a waitlist for key outcomes (Cuijpers et al., 2024, 2025; Monteleone et al., 2022). However, interpreting these findings requires careful consideration of the control condition. The heavy reliance on waitlists presents a significant challenge for accurately estimating CBT's specific efficacy. This finding parallels research on depression, where network meta‐analyses (e.g., Furukawa et al., 2014; Michopoulos et al., 2021) have reported non‐significant differences between CBT and psychological placebo controls—a stark contrast to the large effects observed against waitlist or no‐treatment conditions. These analyses indicate that waitlists consistently function as the “weakest” control, leading to inflated effect sizes. A plausible explanation is that a waitlist fails to control for non‐specific therapeutic factors; patients allocated to this condition receive no clinical attention, which may induce demoralization or a “nocebo” effect.

Regarding head‐to‐head comparisons of CBT with other psychotherapies, previous evidence syntheses are quite inconsistent as relevant trials remain methodologically heterogeneous. In a recent meta‐analysis, Bruns et al. compared CBT to active controls (ranging from psychodynamic, systemic, mindfulness‐based, to more eclectic treatments or psychoeducation) in patients with BN and BED and found no difference regarding eating psychopathology, binge and purging behaviours (Bruns et al., 2025). On the other hand, an umbrella review of meta‐analyses by Monteleone et al. found individual CBT for eating disorders to outperform active controls, where heterogeneous psychotherapies and pharmacological treatments were grouped (Monteleone et al., 2022). This discrepancy may reflect the aggregation of diverse comparators across populations in these evidence syntheses, which appears to be the case in RCTs of psychotherapies: a variety of therapy approaches, each represented by a limited number of studies, mitigate the value of existing conclusions.

In contrast, our focus on active control conditions that may function as a psychological placebo offers a more rigorous benchmark. However, unlike a pharmacologic placebo, a “psychological placebo” is inherently challenging to define and operationalize, typically encompassing heterogeneous interventions that patients perceive as active yet lack a foundation in established psychotherapeutic techniques. In the present synthesis, no original study author explicitly defined an intervention as such; therefore, the self‐help conditions in this study can be viewed as serving this conceptual role by proxy.

Our analysis indicates that when CBT is evaluated against a rigorous comparator—which accounts for non‐specific factors such as a credible rationale and the instillation of hope—its superiority is substantially attenuated across nearly all outcomes. The consistency of this finding suggests that the belief in treatment efficacy itself generates potent therapeutic factors, which likely include enhanced expectation of recovery, a strengthened therapeutic alliance and the general benefit of receiving structured support.

### Limitations

Several limitations of this meta‐analysis warrant consideration. First, and most critically, the evidence base is heavily skewed toward specific comparator types. It relies predominantly on waitlist controls—the weakest comparator, known to inflate effect sizes—while data were scarce for control conditions other than waitlists and self‐help. Crucially, no original study directly compared CBT with an intervention defined as a psychological placebo. Second, heterogeneity presented a challenge. High statistical heterogeneity (*I* ^2^ >60%) was observed for most outcomes, though it was largely resolved in subgroup analyses by control condition. Third, the overall quality of the included RCTs was low, as per our GRADE assessment. All studies were rated as having an unclear or high risk of bias, primarily due to inadequate blinding of participants and personnel—an inherent yet critical methodological challenge in psychotherapy trials. Fourth, the presence of publication bias for the primary outcome of remission, as indicated by small‐study effects (Supplementary Section 9), may have inflated the pooled effect size. Finally, generalizability may be constrained by the stringent eligibility criteria typical of RCTs; thus, the participants may not fully represent the broader clinical population.

## CONCLUSION

In summary, while this meta‐analysis confirms the established superiority of CBT over waitlist controls, this advantage is substantially attenuated when CBT is compared to an active control such as a self‐help intervention. Data for other relevant comparators, including treatment‐as‐usual and pill or psychological placebos, remain scarce. Thus, a more qualified and careful interpretation of CBT's apparent efficacy in the literature—which has been disproportionately informed by comparisons with passive controls—is warranted. To clarify the true contribution of CBT's specific components, future studies should prioritize CBT to two critical benchmarks: rigorous psychological placebo conditions and other structured psychological interventions. Such head‐to‐head trials are essential for isolating the specific therapeutic components and processes fundamental to effectively treating these disorders.

## AUTHOR CONTRIBUTIONS

**Angeliki L. Sioziou:** Data curation; formal analysis; investigation; software; visualization; validation; writing – original draft; writing – review and editing. **Anastasios Stefanou:** Software; data curation; investigation; validation; formal analysis; visualization; writing – original draft; writing – review and editing. **Felicia Dimitrakopoulou:** Writing – original draft; writing – review and editing. **Menelaos Skarlatos:** Data curation; writing – review and editing. **Andreas S. Lappas:** Resources; project administration; methodology; investigation; writing – review and editing. **Christina Mesiari:** Data curation; writing – review and editing. **Aikaterini Argyrou:** Validation; writing – review and editing. **Evangelos Ntouros:** Validation; writing – review and editing. **Nikolaos Christodoulou:** Validation; writing – review and editing. **Vasilios‐Panteleimon Bozikas:** Validation; writing – review and editing. **Myrto Samara:** Conceptualization; methodology; project administration; resources; supervision; writing – review and editing.

## FUNDING INFORMATION

This research did not receive any specific grant from funding agencies in the public, commercial or not‐for‐profit sectors.

## CONFLICT OF INTEREST STATEMENT

Angeliki Siozou, Anastasios Stefanou, Felicia Dimitrakopoulou, Menelaos Skarlatos, Christina Mesiari, Aikaterini Argyrou, Andreas S. Lappas and Nikolaos Christodoulou have no conflicts of interest to disclose. Evangelos Ntouros has received honoraria from Recordati. Vasilios‐Panteleimon Bozikas has received honoraria as a consultant/advisor and/or for satellite symposiums from Johnson and Johnson, Viatris, Vian‐Vianex, Innovis and Teva. MyrtoSamara has received honoraria as a consultant/advisor and/or for lectures from Recordati and Viatris.

## Supporting information


Data S1.

## Data Availability

The R code and associated data files used in this study are available from the authors upon request.
