# Supplementary material for: Cognitive behavioural therapy compared to control conditions in treating bulimia nervosa and binge‐eating disorder: A systematic review and meta‐analysis
Source: Br J Clin Psychol. 2026 May 22;65(4):1248–61. doi: 10.1111/bjc.70070 (PMC13655980; doi:10.1111/bjc.70070)
Supplement: Supplementary file 1 [file BJC-65-1248-s001.docx]

SUPPLEMENTARY MATERIAL

Table of Contents

[SUPPLEMENT 1: Protocol of the systematic review and meta-analysis	2](#_Toc227936140)

[SUPPLEMENT 2: PRISMA checklist	6](#_Toc227936141)

[SUPPLEMENT 3. Search Strategy	9](#_Toc227936142)

[SUPPLEMENT 4. PRISMA Flow Diagram	12](#_Toc227936143)

[SUPPLEMENT 5. Included studies	13](#_Toc227936144)

[SUPPLEMENT 6: Risk of bias assessment in included studies	41](#_Toc227936145)

[SUPPLEMENT 7: Forest plots for all outcomes	44](#_Toc227936146)

[SUPPLEMENT 8: Meta-regression	65](#_Toc227936147)

[SUPPLEMENT 9: Publication bias	74](#_Toc227936148)

[SUPPLEMENT 10: GRADE	78](#_Toc227936149)

## **SUPPLEMENT 1: Protocol of the systematic review and meta-analysis**

Review question

The objective of this study is to systematically review the literature and to conduct a meta-analysis comparing the effects of cognitive behavioral therapy (CBT) to control conditions for patients with bulimia nervosa and binge eating disorder.

Searches

The following sources will be searched:

1. Electronic databases: Multiple systematic searches will be conducted using databases such as EMBASE, PubMed, Cochrane Central Register of Controlled Trials (CENTRAL) and ClinicalTrials.gov
2. Previous reviews: we will search previous reviews for relevant studies.
3. Reference searching: we will inspect the references of all identified studies for more trials.
4. Multiple systematic searches will be conducted using databases such as PubMed and EMBASE, the database of the Cochrane group “, and ClinicalTrials.gov. The search will comprise terms related to randomized trials, bulimia nervosa and binge eating disorder.

There will be no limitation about language, date and publication status of the studies.

Types of study to be included

We will include all the relevant Randomized Controlled Trials (RCTs) in order to assess the efficacy of psychotherapy in bulimia nervosa and binge eating disorder.

Condition or domain being studied

Bulimia nervosa or binge eating disorder, irrespective of the diagnostic system applied.

Participants/population

People with bulimia nervosa or binge eating disorder. There will be no restriction concerning age, gender and comorbidities.

Intervention(s), exposure(s)

Standard individual or group CBT, online or in-person.

Comparator(s)/control

Guided or unguided self-help interventions, placebo (psychological or pill), waiting list, no treatment or any other form of no treatment.

Primary outcome(s)

The main outcomes will be:

1. Remission: 100% reduction in binge eating-related symptoms as no longer meeting the relevant diagnostic criteria (including cognitive elements).

2. Frequency of binges

Measures of effect

Risk Ratio, Mean Difference, Standardized Mean Difference

Secondary outcome(s)

1) Frequency of purges

2) Response: a reduction of at least 50% in bulimic/binge-eating episodes. But as these may be rarely indicated, we will also use the authors' definitions of response. If many definitions of response are provided, we will select the strictest.

3) Eating disorder psychopathology measured by Eating Disorder Inventory (EDI) or any other validated scale.

4) Depression measured by rating scales such as Hamilton Depression Rating Scale (HDRS), Beck Depression Inventory (BDI) or any other validated scale.

5) Anxiety measured by rating scales such as Hamilton Anxiety Rating Scale (HAM-A) or any other validated scale.

6) Self esteem

7) Weight (kg)

8) BMI (kg/m2)

9) Quality of life (QoL) measured by rating scales such as The Quality of Life Scale (QOLS). The improvement of patients’ wellbeing or change in quality of life are significant goals for psychiatric care.

10) Total dropouts

11) Dropouts due to side effects

12) Total number of patients with adverse effects

13) Specific adverse effects

Measures of effect

Risk Ratio, Mean Difference, Standardized Mean Difference

Data extraction (selection and coding)

Two review authors will independently extract data from all included studies. Any disagreement will be discussed with a third author. If the disagreement is not solved we will try to have a contact with the authors of the study for clarification. Change scores will be preferred over endpoint values.

Risk of bias (quality) assessment

Two review authors will work independently to assess the risk of bias by using the Cochrane Risk of Bias Tool for Randomized Trials (RoB).

Strategy for data synthesis

We will employ a random-effects model for analyses (Der-Simonian 1986). We understand that there is no closed argument for preference of fixed or random effects model. The random-effects model is usually more conservative in terms of statistical significance, although as a disadvantage it puts added weight onto smaller studies which can either inflate or deflate the effect size. Therefore, we will examine in a sensitivity analysis whether using a fixed-model markedly changes the results of the primary outcomes.

All analyses will be on an intention-to-treat (ITT) basis. We will also conduct a sensitivity analysis by excluding studies which presented only completer data.

The effect size for dichotomous outcomes will be Risk Ratio (RR). The effect size for continuous outcomes will be weighted mean difference (MD). If data are presented in different scales, then the standardized mean difference (SMD) will be calculated instead. Missing standard deviations will be calculated from standard errors or estimated from confidence intervals, t-values, or p-values as described in Section 7.7.3 of the Cochrane Handbook for Systematic Reviews.

Heterogeneity: All included trials will be considered initially. Heterogeneity will be investigated by inspection of the forest plots. Statistical heterogeneity will be tested with the χ² test and quantified by the I² statistics and its 95% confidence interval (CI). Potential reasons for heterogeneity will be explored with subgroup analyses, but these will only be conducted on the primary outcomes.

Analysis of subgroups or subsets

Subgroup and sensitivity analyses will be conducted on the primary outcomes, namely response, frequency of purges and eating disorder psychopathology.

Subgroup analysis will be conducted :

a) CBT vs each different control condition.

b) People with bulimia nervosa and people with binge eating disorder

c) Previously treated participants versus not

d) Participants with comorbidities versus no comorbidities

e) Allegiance bias, i.e. whether the inventors of a psychotherapy are also the authors of a trial, because this could lead to bias

f) Attrition bias

g) Detection bias

h) Duration of psychotherapy up to 6 weeks versus longer

i) Duration of psychotherapy (continuous)

j) Baseline severity (continuous)

Sensitivity analyses:

1) Exclusion of non-double- blind studies (open and single-blind studies)

2) Exclusion of studies that presented only completer analyses

3) Exclusion of studies with high risk of bias

4) Fixed effects instead of random effects model

1.1 Deviations from protocol

Prompted by clinical interest, we also performed a subgroup analysis per format of CBT, (i.e. group or individual therapy). We present these results as exploratory.

## **SUPPLEMENT 2: PRISMA checklist**

**Table S1.** PRISMA Checklist

| **Section /Topic** | **Item #** | **Checklist item** | **Reported on Page #** |
| --- | --- | --- | --- |
| **TITLE** | | | |
| **Title** | 1 | Identify the report as a systematic review, meta-analysis, or both. . | 1-2 |
| **ABSTRACT** | | | |
| **Abstract** | 2 | Provide a structured summary including, as applicable: background; objectives; data sources; study eligibility criteria, participants, and interventions; study appraisal and synthesis methods; results; limitations; conclusions and implications of key findings; systematic review registration number. | 3-4 |
| **INTRODUCTION** | | | |
| **Rationale** | 3 | Describe the rationale for the review in the context of what is already known. | 5-6 |
| **Objectives** | 4 | Provide an explicit statement of questions being addressed with reference to participants, interventions, comparisons, outcomes, and study design (PICOS). | 6 |
| **METHODS** | | | |
| **Protocol and registration** | 5 | Indicate if a review protocol exists, if and where it can be accessed (e.g., Web address), and, if available, provide registration information including registration number. | 6-7 & supp 1 |
| **Eligibility criteria** | 6 | Specify study characteristics (e.g., PICOS, length of follow-up) and report characteristics (e.g., years considered, language, publication status) used as criteria for eligibility, giving rationale. | 7-8 |
| **Information sources** | 7 | Describe all information sources (e.g., databases with dates of coverage, contact with study authors to identify additional studies) in the search and date last searched. | 8 & supp 4 |
| **Search selection** | 8 | Present full electronic search strategy for at least one database, including any limits used, such that it could be repeated. | supp 4 |
| **Study selection** | 9 | Specify the methods used to decide whether a study met the inclusion criteria of the review, including how many reviewers screened each record and each report retrieved, whether they worked independently, and if applicable, details of automation tools used in the process. | 8 |
| **Data collection process** | 10 | Describe method of data extraction from reports (e.g., piloted forms, independently, in duplicate) and any processes for obtaining and confirming data from investigators. | 9-10 |
| **Data items** | 11 | List and define all variables for which data were sought (e.g., PICOS, funding sources) and any assumptions and simplifications made. | 7-8 |
| **Risk of bias in individual studies** | 12 | Describe methods used for assessing risk of bias of individual studies (including specification of whether this was done at the study or outcome level), and how this information is to be used in any data synthesis. | 8 & supp 7 |
| **Summary measures** | 13 | State the principal summary measures (e.g., risk ratio, difference in means). | 10 |
| **Synthesis of results** | 14 | Describe the methods of handling data and combining results of studies, if done, including measures of consistency (e.g., I^2^) for each meta-analysis. | 9-10 |
| **Risk of bias across studies** | 15 | Specify any assessment of risk of bias that may affect the cumulative evidence (e.g., publication bias, selective reporting within studies). | 10 |
| **Additional analyses** | 16 | Describe methods of additional analyses (e.g., sensitivity or subgroup analyses, meta-regression), if done, indicating which were pre-specified. | 9-10 |
| **RESULTS** | | | |
| **Study selection** | 17 | Give numbers of studies screened, assessed for eligibility, and included in the review, with reasons for exclusions at each stage, ideally with a flow diagram. | 11; supp 5 |
| **Study characteristics** | 18 | For each study, present characteristics for which data were extracted (e.g., study size, PICOS, follow-up period) and provide the citations. | 11 and supp 6 |
| **Risk of bias in studies** | 19 | Present data on risk of bias of each study and, if available, any outcome level assessment (see item 12). | 11 and supp 7 |
| **Results of individual studies** | 20 | For all outcomes considered (benefits or harms), present, for each study: (a) simple summary data for each intervention group (b) effect estimates and confidence intervals, ideally with a forest plot. | 11-14 and supp 9 |
| **Synthesis of results** | 21 | Present results of each meta-analysis done, including confidence intervals and measures of consistency. | 11-14 and supp 9 |
| **Risk of bias across studies** | 22 | Present results of any assessment of risk of bias across studies (see Item 15). | 15 and supp 9 |
| **Additional analysis** | 23 | Give results of additional analyses, if done (e.g., sensitivity or subgroup analyses, meta-regression [see Item 16]). | 14-15 and supp 7-8 |
| **DISCUSSION** | | | |
| **Discussion** | 24 | Summarize the main findings including the strength of evidence for each main outcome; consider their relevance to key groups (e.g., healthcare providers, users, and policy makers). | 15-18 |
| **Limitations** | 25 | Discuss limitations at study and outcome level (e.g., risk of bias), and at review-level (e.g., incomplete retrieval of identified research, reporting bias). | 17-18 |
| **Conclusions** | 26 | Provide a general interpretation of the results in the context of other evidence, and implications for future research. | 18 |
| **FUNDING** | | | |
| **Funding** | 24a | Describe sources of funding for the systematic review and other support (e.g., supply of data); role of funders for the systematic review. | 18 |

## **SUPPLEMENT 3. Search Strategy**

The search strategy for Ovid Medline is detailed below. Variations of this search strategy were tailored to meet the specific requirements of the additional databases, including (1) APA PsycInfo, (2) Cochrane Central Register of Controlled Trials (CENTRAL), and (3) Embase. APA PsychInfo, Embase and Medline were searched via Ovid. Additionally, the WHO International Clinical Trials Registry Platform (ICTRP) and the clinical trials register ClinicalTrials.gov were searched individually, using DORAs and all their variations as a keyword.

**Table S2.** Ovid MEDLINE Search String.

**1 ("no treatment" or "usual care" or "treatment as usual" or "care as usual" or "non-intervention" or "standard care" or "monitoring only" or "observation only" or "psychological placebo*" or "mental placebo*" or "psychological control*" or "placebo response*" or "placebo effect*" or "supportive therapy" or "non-directive counsel*" or "minimal intervention" or "attention control" or "stress management" or "relaxation" or "educational intervention" or "befriending therapy" or "placebo psychotherapy" or "general supportive discussion" or "psychoeducation-only intervention*" or "sham therapy" or "activity-based placebo*" or "lifestyle advice" or "relaxation training" or "neutral task assignment*" or "therapist check-in*" or "diet management" or "health education" or "basic nutritional counsel*" or "guided self-help" or "self-monitoring" or "neutral goal setting" or "symptom monitoring" or "mindfulness exercises" or "support group" or "structured journaling").tw,kf. (327860)**

**2 psychological placebo.mp. (63)**

**3 1 or 2 (327860)**

**4 randomized controlled trial.pt. (630596)**

**5 controlled clinical trial.pt. (95679)**

**6 randomized.ab. (677409)**

**7 placebo.ab. (255502)**

**8 drug therapy.fs. (2773987)**

**9 randomly.ab. (451622)**

**10 trial.ab. (734798)**

**11 groups.ab. (2797173)**

**12 or/4-11 (6184468)**

**13 exp animals/ not humans.sh. (5299911)**

**14 12 not 13 (5417981)**

**15 3 and 14 (115020)**

**16 exp Bulimia Nervosa/ or exp Bulimia/ or bulimia.mp. (12620)**

**17 binge eating disorder.mp. or exp Bulimia/ or exp Binge-Eating Disorder/ (9720)**

**18 (purge or purging).tw,kf. (7889)**

**19 (binge or bingeing or binging).tw,kf. (19230)**

**20 bulimia.tw,kf. (9202)**

**21 or/16-20 (32724)**

**22 15 and 21 (391)**

## **SUPPLEMENT 4. PRISMA Flow Diagram**

**Figure S1.** PRISMA Flow Diagram

**Identification of studies via databases and registers**

Records removed *before screening*:

- Duplicate records removed (n = 227)
- Records markers as ineligible by automation tools (n = 0)
- Records removed for other reasons (n = 0)

Records identified through databases and registries searching up to December 2025 (n = 5144)

**Identification**

Records excluded as irrelevant

(n = 719)

Records screened (n = 4977)

Reports not retrieved

(n=948)

Reports sought for retrieval (n=4258)

- Comparison not relevant (n = 0)
- Outcomes not relevant (n = 209)
- Population not relevant (n = 2041)
- Intervention not relevant (n = 163)
- Study design not relevant (n = 855)

**Screening**

Reports excluded with reasons (n=3268):

- Comparison not relevant (n = 0)
- Outcomes not relevant (n = 209)
- Population not relevant (n = 2041)
- Intervention not relevant (n = 163)
- Study design not relevant (n = 855)

Reports assessed for eligibility (n=3310)

Reports of included studies

(n=42)

**Included**

## **SUPPLEMENT 5.** **Included studies**

**Table S3.** Characteristics of Included studies

| **First author, publication, date** | | **Study design** | | | **Duration (weeks)** | | | **Participants mean age (year)** | | | **N of randomized patients on CBT and number of sessions** | | | **N of randomized patients on self- help, placebo, treatment as usual or no treatment (Control)** | | | | | **Baseline weight/sd(range)-BMI/sd(range)** | **Type of CBT** | | | | | **Study country** | **Definition of remission/ response to treatment** | | | **Diagnosis/**  **Diagnostic Criteria** | | |
| --- | --- | --- | --- | --- | --- | --- | --- | --- | --- | --- | --- | --- | --- | --- | --- | --- | --- | --- | --- | --- | --- | --- | --- | --- | --- | --- | --- | --- | --- | --- | --- |
|  | **Waiting list** | | | | | | | | | | | | | | | | | | | | | | | | | | | | | | |
| **Agras 1989** (Agras et al., 1989) | | Randomized Controlled Trial/Parallel | | | 16 | | | 29.2 | | | N=22  14 sessions | | | **Wlt:** N=19 | | | | | n.i | Individual CBT | | | | | USA | n.i | | | BN/  DSM-III-R | | |
| **Agras 1995** (Agras et al., 1995) | | Randomized Controlled Trial/Parallel | | | 12 | | | 47.6 | | | Ν= 39  12 sessions | | | **Wlt**: N=11 | | | | | CBT  weight: 108 kg/26.7 (91.9-108)  Wlt  weight: 106.1 kg/20.3 (95.42-111) | Group CBT | | | | | USA | Abstinent from binge episodes the past 2 weeks (remission – response) | | | BED/  DSM-IV | | |
| **Allen 1999** (Allen N. Heather; & Craighead Wilcoxon Linda, 1999) | | Randomized Controlled Trial/Open/ Parallel | | | 8 | | | 21 | | | Ν= 15  8 sessions | | | **Wlt**: N=14 | | | | | n.i | Group CBT | | | | | USA | n.i | | | BED/  DSM-IV | | |
| **Chao 2024** (Chao et al., 2024) | | Randomized Controlled Trial/Parallel | | | 16 | | | 29.8 | | | N=20 | | | **Wlt**: N=20 | | | | | CBT  BMI: 36.08 kg/m^2^/7.5(20.7-42.59)  Wlt  BMI:35.21 kg/m^2^ /7.09 (21.7-42.58) | Individual CBT | | | | | USA | No binge episodes during last 4 weeks (remission) | | | BED/ DSM-V | | |
| **Davis 1998** (Davis et al., 1998) | | Randomized Controlled Trial/Parallel | | | 16 | | | 27.1 | | | Ν= 39  16 sessions | | | **Wlt**: N=19 | | | | | n.i | Group CBT | | | | | Canada | Complete remission for binging and purging (response) | | | BN/  DSM-III-R | | |
| **Debar 2013** (DeBar et al., 2013) | | Randomized Controlled Trial/Parallel | | | 24 | | | 15.1 | | | Ν= 13  12 sessions | | | **Wlt**: N=13 | | | | | n.i | Individual CBT | | | | | USA | Abstinent of ΟΒΕs in 3 or 6 months (remission - response) | | | BED/  DSM-IV | | |
| **Dingemans 2007** (Dingemans et al., 2007) | | Randomized Controlled Trial/Open/ Parallel | | | 20 | | | n.i | | | Ν=30  15 sessions | | | **Wlt**: N=22 | | | | | n.i | Group CBT | | | | | The Netherlands | Dropped frequency of OBEs the previous 28 days (response) | | | BED /  DSM-IV | | |
| **Eldrerge 1996** (Eldredge et al., 1997) | | Randomized Controlled Trial/Parallel | | | 12 | | | 45.2 | | | Ν= 36  12 sessions | | | **Wlt**: N=10 | | | | | n.i | Group CBT | | | | | USA | Abstinence from binge eating (response) | | | BED/  DSM-IV | | |
| **Freeman 1988** (Freeman et al., 1988) | | Randomized Controlled Trial/Parallel | | | 15 | | | 24.2 | | | Ν=62  15 sessions | | | Wlt: N=20 | | | | | n.i | Group CBT | | | | | UK | n.i | | | BN/  DSM-III | | |
| **Gorin 2002** (Gorin et al., 2002) | | Randomized Controlled Trial/Open/ Parallel | | | 12 | | | 45.2 | | | Ν= 63  12 sessions | | | Wlt: N=61 | | | | | CBT  BMI: 39.63 kg/m^2^ /8.55(20.7-42.59)  Wlt  BMI: 39.37 kg/m^2^/7.53 (21.7-42.58) | Group CBT | | | | | USA | Binge abstinence at the end of treatment (response) | | | BED/  DSM-IV | | |
| **Griffiths 1994** (Griffiths et al., 1994) | | Randomized Controlled Trial/Parallel | | | 8 | | | 25.9 | | | Ν=20  8 sessions | | | Wlt: N=22 | | | | | n.i | Individual CBT | | | | | USA | Abstinent from bingeing (response) | | | BN/  DSM-III-R | | |
| **Hilbert 2019** (Hilbert et al., 2019) | | Randomized Controlled Trial/Open/ Parallel | | | 16 | | | 15.3 | | | Ν= 37  20 sessions | | | Wlt: N=36 | | | | | n.i. | Individual CBT | | | | | Germany | Don’t meet diagnostic criteria for at least 3 months (remission)/Abstinence from binge eating over the past 28 days (response) | | | BED/  DSM-IV-TR, DSM-V | | |
| **Laessle 1987** (Laessle et al., 1987) | | Randomized Controlled Trial/Parallel | | | 16 | | | n.i | | | Ν= 8  24 sessions | | | Wlt: N=9 | | | | | CBT  BMI: 20.8 kg/m^2^/2.3 (20.7-42.59)  Wlt  BMI: 21.8 kg/m^2^/1.9 (21.7-42.58) | Group CBT | | | | | Germany | Abstinent from binging (response) | | | BN/  DSM-III | | |
| **Lee 1986** (Lee & Rush, 1986) | | Randomized Controlled Trial/Parallel | | | 6 | | | 27.7 | | | Ν= 15  12 sessions | | | Wlt: N=15 | | | | | n.i | Group CBT | | | | | USA | At least a 50% decline in binge frequency from pretreatment to posttreatment (response) | | | BN/  DSM-III | | |
| **Leitenberg 1988** (Leitenberg et al., 1988) | | Randomized Controlled Trial/Parallel | | | 14 | | | 26 | | | Ν= 35  14 sessions | | | Wlt: N=12 | | | | | n.i | Group CBT | | | | | USA | n.i | | | BN/  DSM-II | | |
| **Lewer 2017** (Lewer et al., 2017) | | Randomized Controlled Trial/Open/ Parallel | | | 10 | | | n.i | | | Ν= 15  10 sessions | | | Wlt: N=19 | | | | | n.i | Group CBT | | | | | Germany | n.i | | | BED/  DSM-IV | | |
| **Mathisen 2018** (Mathisen et al., 2018) | | Randomized Controlled Trial/Open/ Parallel | | | 16 | | | n.i | | | Ν= 78 (51BN,27BED)  20 sessions | | | Wlt: N=23 (2O BN,3 BED) | | | | | n.i | Group CBT | | | | | Norway | n.i | | | BN/BED  DSM-V | | |
| **Peterson 1997** (Peterson et al., 1997) | | Randomized Controlled Trial/Parallel | | | 8 | | | 42.4 | | | Ν= 16  8 sessions | | | Wlt: N=11 | | | | | n.i | Group CBT | | | | | USA | Abstinence rate from objective binge episodes the last 8 weeks (remission – response) | | | BED/  DSM-IV | | |
| **Peterson 2009** (Peterson et al., 2009) | | Randomized Controlled Trial/Parallel | | | 20 | | | 47.1 | | | Ν=60  20 sessions | | | Wlt: N=69 | | | | | CBT  BMI: 39.73 kg/m^2^/8.49 (20.7-42.59)  Wlt  BMI: 38.1 kg/m^2^/6.9 (21.7-42.58) | Group CBT | | | | | USA | Abstinence of binge eating episodes previous 28 days (remission – response) | | | BED/  DSM-IV | | |
| **Schlup 2009** (Schlup et al., 2009) | | Randomized Controlled Trial/Open/ Parallel | | | 8 | | | 44.3 | | | Ν= 18  16 sessions | | | Wlt: N=18 | | | | | CBT  BMI: 32.4 kg/m^2^/5.6 (20.7-42.59)  Wlt  BMI: 34.3 kg/m^2^/9.1 (21.7-42.58) | Group CBT | | | | | Switzerland | Abstinence from binge eating (zero OBEs in the past 28 days) (response-remission) | | | BED /  DSM-IV-TR, DSM-V | | |
| **Shapiro 2007** (Shapiro et al., 2007) | | Randomized Controlled Trial/Parallel | | | 10 | | | 39.6 | | | Ν= 22  10 sessions | | | Wlt: N=22 | | | | | CBT  BMI: 37.72 kg/m^2^/9.45 (20.7-42.59)  Wlt  BMI: 34.7 kg/m^2^/6.61 (21.7-42.58) | Group CBT | | | | | USA | Abstinence of binge eating episodes (response) | | | BED/  DSM-IV | | |
| **Shelley-Ummenhofer 2007**(Shelley-Ummenhofer & MacMillan, 2007) | | Randomized Controlled Trial/Parallel | | | 6 | | | N.I | | | Ν= 13  6 sessions | | | Wlt: N=9 | | | | | CBT  weight: 107.2 kg/17.28 (91.9-108)  Wlt  Weight: 102.52 kg/14.7 (95.42-111) | Group CBT | | | | | Canada | n.i | | | BED/  DSM-IV-TR | | |
| **Sundgot -Borgen 2002** (Sundgot-Borgen et al., 2002) | | Randomized Controlled Trial/Parallel | | | 16 | | | 16 | | | Ν= 16  16 sessions | | | Wlt: N=16 | | | | |  | Group CBT | | | | | Norway | n.i | | | BN/  DSM-IV | | |
| **Tasca 2007** (Tasca et al., 2007) | | Randomized Controlled Trial/Parallel | | | 16 | | | 42.8 | | | Ν=47  16 sessions | | | Wlt: N=40 | | | | | CBT  BMI: 42.59 kg/m^2^/12.95 (20.7-42.59)  Wlt  BMI: 42.58 kg/m^2^/9.57 (21.7-42.58) | Group CBT | | | | | Canada | Improved binge symptoms (response) | | | BED/  DSM-IV | | |
| **Telch 1990** (Telch et al., 1990) | | Randomized Controlled Trial/Parallel | | | 10 | | | 42.6 | | | Ν= 23  10 sessions | | | Wlt: N=21 | | | | | n.i | Group CBT | | | | | USA | Reduction of binge episodes/weekly (response) | | | BN/  DSM-III-R | | |
| **ter Huurne 2015** (Ter Huurne et al., 2015) | | Randomized Controlled Trial/Parallel | | | 15 | | | 39.4 | | | Ν= 108  30 sessions | | | Wlt: N=106 | | | | | n.i | Individual CBT | | | | | The Netherlands | n.i | | | BN/BED  DSM-IV | | |
| **Treasure 1994** (Treasure et al., 1994) | | Randomized Controlled Trial/Parallel | | | 16 | | | N.I | | | Ν= 21  16 sessions | | | Wlt: N=19 | | | | | CBT  BMI: 26.8 kg/m^2^/7 (20.7-42.59)  Wlt  BMI: 23.3 kg/m^2^/6.7 (21.7-42.58) | Individual CBT | | | | | UK | Abstinence from binge/purge after 8 weeks of treatment (response) | | | BN/  ICD-10 | | |
| **Wagner 2016** (Wagner et al., 2016) | | Randomized Controlled Trial/Parallel | | | 16 | | | 35.1 | | | Ν= 69  16 sessions | | | Wlt: N=70 | | | | | CBT  weight: 91.9 kg/21 (91.9-108)  BMI: 31.9 kg/m^2^/6.7 (20.7-42.59)  Wlt  weight: 95.42 kg/24.1 (95.42-24.1)  BMI: 32.8 kg/m^2^/8.1 (21.7-42.5 | Individual CBT | | | | | Germany | Less than 4 OBEs after treatment (response) | | | BED/  DSM-IV | | |
| **Wilfley 1993** (Wilfley et al., 1993) | | Randomized Controlled Trial/Parallel | | | 16 | | | 44.3 | | | Ν= 18  16 sessions | | | Wlt: N=20 | | | | | n.i | Group CBT | | | | | USA | Reduction of binge episodes (response) | | | BN/  DSM-III-R | | |
| **Wolf 1992** (Wolf & Crowther, 1992) | | Randomized Controlled Trial/Open/ Parallel | | | 8 | | | 26 | | | Ν= 30  10 sessions | | | Wlt: N=11 | | | | | n.i | Group CBT | | | | | USA | n.i | | | BN/  DSM-III | | |
|  | **Self-help (guided/unguided)** | | | | | | | | | | | | | | | | | | | | | | | | | | | | | | |
| **Agras 1989** (Agras et al., 1989) | | | | Randomized Controlled Trial/Parallel | | | 16 | | | 29.2 | | | N=22  14 sessions | | | Guided Self-help **(**CBT- based**;** self-monitoring of caloric intake and purging behaviors): N=19 | | **n.i** | | | | Individual CBT | | USA | | | | n.i | | | BN/  DSM-III-R |
| **Bailer 2004** (Bailer et al., 2004) | | | | Randomized Controlled Trial/Open/ Parallel | | | 18 | | | n.i | | | Ν=41  18 sessions | | | Guided Self-help (guided self-help manual with a maximum of 18 short weekly visits): N=40 | | CBT  BMI: 20.7 kg/m^2^/2.4 (20.7-42.59)  GSH  BMI: 21.7 kg/m^2^/3.1 (21.7-42.58) | | | | Group CBT | | Austria | | | | Abstinent from binge & purge episodes the month before assessment (remission-response) | | | BN/  DSM-IV |
| **deZwaan 2017** (De Zwaan et al., 2017) | | | Randomized Controlled Trial/Parallel | | | 16 | | | | 43.2 | | | N=86  20 sessions | | | Guided Self-help (Internet-based CBT): N=83 | | CBT  BMI: 34.4 kg/m^2^/3.9 (20.7-42.59)  GSH  BMI: 33.4 kg/m^2^/3.9 (21.7-42.58) | | | | Individual CBT | | Germany | | | | No binge episodes during last 4 weeks (remission) | | | BED/ DSM-IV-TR |
| **Hsu 2001** (Hsu et al., 2001) | | | Randomized Controlled Trial/Parallel | | | 14 | | | 24.5 | | | N=26  16 sessions | | | Unguided Self-help: N=24 | | n.i | | | | Individual CBT | | USA | | | | No binge/vomit episode the week before post-treatment assessment at week 14 (response-remission) | | | BN/ DSM-III-R | |
| **Kenardy 2002** (Kenardy et al., 2002) | | | Randomized Controlled Trial/Open/ Parallel | | | 10 | | | n.i. | | | N=17  10 sessions | | | Unguided Self-help (NPT: non-prescriptive therapy-non-directive counselling and focused evocative unfolding): N=17 | | CBT  BMI: 40.18 kg/m^2^/8.46 (20.7-42.59)  UnGSH  BMI: 35.49 kg/m^2^/5.97 (21.7-42.58) | | | | Group CBT | | Australia | | | | n.i. | | | BED/ Oxford criteria for BED | |
| **Kirkley 1985** (Kirkley et al., 1985) | | | Randomized Controlled Trial/Parallel | | | 16 | | | 28.3 | | | N=14 | | | Unguided Self-help (group based self-monitoring-non-directive): N=14 | | n.i | | | | Group CBT | | USA | | | | n.i. | | | BN/  DSM-III | |
| **Peterson 2009** (Peterson et al., 2009) | | | Randomized Controlled Trial/Parallel | | | 20 | | | 47.1 | | | Ν=60  20 sessions | | | Unguided Self-help  **N=67** | | CBT  BMI: 39.73 kg/m^2^/8.49 (20.7-42.59)UnGSH  BMI: 38.2 kg/m^2^/7.2 (21.7-42.58) | | | | Group CBT | | USA | | | | Abstinence of binge eating episodes previous 28 days (remission – response) | | | BED/  DSM-IV | |
| **Thackwray 1993** (Thackwray et al., 1993) | | | Randomized Controlled Trial/Parallel | | | 8 | | | 31.3 | | | N= 16  8 sessions | | | Unguided Self-help (NSMT: nonspecific self-monitoring treatment): N=16 | | n.i | | | | Individual CBT | | USA | | | | n.i. | | | BN/ DSM-III | |
| **Treasure 1994** (Treasure et al., 1994) | | | Randomized Controlled Trial/Parallel | | | 16 | | | N.I | | | Ν= 21  16 sessions | | | Unguided Self-help  N=41 | | CBT  BMI: 26.8 kg/m^2^/7 (20.7-42.59)  UnGSH  BMI: 24 kg/m^2^/5.9 (21.7-42.58) | | | | Individual CBT | | UK | | | | Abstinence from binge/purge after 8 weeks of treatment (response) | | | BN/  ICD-10 | |
| **Zerwas 2017**(Zerwas et al., 2016) | | | Randomized Controlled Trial/Parallel | | | 20 | | | n.i. | | | N=90  16 sessions | | | Guided Self-help (Internet-based CBT): N=89 | | **n.i** | | | | Group CBT | | USA | | | | Zero binge episodes the last 28 days (remission-response) | | | BN/ DSM-IV | |

|  | **TAU (treatment as usual)** | | | | | | | | | | |
| --- | --- | --- | --- | --- | --- | --- | --- | --- | --- | --- | --- |
| **Cesa 2013** (Cesa et al., 2013) | | Randomized Controlled Trial/Parallel | 5 | 31.8 | Ν=47  (27-ECT & 20 CBT)  15 sessions | IP:The Integrated Multimodal Medically Managed Inpatient) : N=19 | CBT  weight: 106.6 kg/8.9 (91.9-108)  BMI:41.1 kg/m^2^/3.3 (20.7-42.59)  IP  weight: 103 kg/18.2 (95.42-111)  BMI: 39.2 kg/m^2^/5.3 (21.7-42.58) | Group (5) & Individual (10) CBT | Italy | No binge episodes during treatment (remission-response) | BED/  DSM-IV-TR |

|  | **Pill placebo** | | | | | | | | | | |
| --- | --- | --- | --- | --- | --- | --- | --- | --- | --- | --- | --- |
| **Devlin**  (Devlin et al., 2005) | | Randomized Controlled Trial/Parallel | 20 | 43 | N=25  20 sessions | Pill placebo: N=31 | CBT  Weight: 116kg/ 22.2  BMI: 41.1 kg/m^2^/ 7.6  Pill pbo  Weight: 113.5kg / 22.2  BMI: 40.3 kg/m^2^/ 7.1 | Individual CBT | USA | No binge eating during last 4weeks (remission) | BED/  EDE-BED |
| **Grilo 2005** (Grilo et al., 2005) | | Randomized Controlled Trial/Parallel | 16 | 44 | N=28  16 sessions | Pill placebo: N=27 | CBT  BMI: 35 kg/m^2^/6.2 (20.7-42.59)  Pill pbo  BMI: 35.7 kg/m^2^/7.2 (21.7-42.58) | Individual CBT | USA | No binge episodes during last 4 weeks (remission) | BED/ DSM-IV |
| **Mitchell 1990** (Mitchell et al., 1990) | | Randomized Controlled Trial/Parallel | 10 | n.i | N=34  (Structured Intensive Psychotherapy)  28 sessions | Pill placebo: N=31 | n.i | Group CBT | USA | n.i. | BN/  DSM-III |

|  | | **No treatment** | | | | | | | | | | | | |
| --- | --- | --- | --- | --- | --- | --- | --- | --- | --- | --- | --- | --- | --- | --- |
| **Schag 2019** (Schag et al., 2019) | | | | Randomized Controlled Trial/Parallel | 8 | n.i. | Ν= 41  (IMPULS)  8 sessions | No treatment: N=39 | CBT  BMI: 36.2 kg/m^2^/8.9 (20.7-42.59)  No treatment  BMI: 37.7 kg/m^2^/10.2 (21.7-42.58) | Group CBT | Germany | No binge eating episodes in the past 4 weeks (remission) | BED/ DSM-V | |
|  |  | | **CBT**: *Cognitive – Behavioral Therapy,* **BN**: *Bulimia Nervosa,* **BED**: *Binge Eating Disorder,* **EDE:** Eating Disorder Examination, **EDNOS:** Eating Disorder Not Otherwise Specified, **DSM**: *Diagnostic and Statistical Manual,* **ICD**: *International Classification of Diseases,* **n.i**: *not indicated* | | | | | | | | | | |  |

References for Included studies

Agras, W. S., Schneider, J. A., Arnow, B., Raeburn, S. D., & Telch, C. F. (1989). Cognitive-Behavioral and Response-Prevention Treatments for Bulimia Nervosa. *Journal of Consulting and Clinical Psychology*, *57*(2), 215–221. https://doi.org/10.1037/0022-006X.57.2.215

Agras, W. S., Telch, C. F., Arnow, B., Eldredge, K., Detzer, M. J., Henderson, J., Marnell, M., Agras, W. S., Telch, C. F., Arnow, B., Eldredge, K., Detzer, M. J., Henderson, J., & Marnell, M. (1995). *Does Interpersonal Therapy Help Patients With Binge Eating Disorder Who Fail to Respond to Cognitive-Behavioral Therapy ?* *63*(3), 356–360.

Allen N. Heather;, & Craighead Wilcoxon Linda. (1999). *Appetite Monitoring in the Treatment of Binge Eating Disorder*. 253–272.

Bailer, U., De Zwaan, M., Leisch, F., Strnad, A., Lennkh-Wolfsberg, C., El-Giamal, N., Hornik, K., & Kasper, S. (2004). Guided Self-Help Versus Cognitive-Behavioral Group Therapy in the Treatment of Bulimia Nervosa. *International Journal of Eating Disorders*, *35*(4), 522–537. https://doi.org/10.1002/eat.20003

Cesa, G. L., Manzoni, G. M., Bacchetta, M., Castelnuovo, G., Conti, S., Gaggioli, A., Mantovani, F., Molinari, E., Cárdenas-López, G., & Riva, G. (2013). Virtual reality for enhancing the cognitive behavioral treatment of obesity with binge eating disorder: Randomized controlled study with one-year follow-up. *Journal of Medical Internet Research*, *15*(6). https://doi.org/10.2196/jmir.2441

Chao, A. M., Agarwal, K., Zhou, Y., Grilo, C. M., Gur, R. C., Joseph, P., Shinohara, R. T., Richmond, T. S., & Wadden, T. A. (2024). Neural Responses to Auditory Food Stimuli Following Cognitive Behavioral Therapy for Binge-Eating Disorder. *International Journal of Eating Disorders*, *57*(9), 1911–1923. https://doi.org/10.1002/eat.24244

Davis, R., Mcvey, G., Heinmaa, M., Rockert, W., & Kennedy, S. (1998). *Sequencing of Cognitive-Behavioral Treatments for Bulimia Nervosa*.

De Zwaan, M., Herpertz, S., Zipfel, S., Svaldi, J., Friederich, H. C., Schmidt, F., Mayr, A., Lam, T., Schade-Brittinger, C., & Hilbert, A. (2017). Effect of internet-based guided self-help vs individual face-to-face treatment on full or subsyndromal binge eating disorder in overweight or obese patients: The INTERBED randomized clinical trial. *JAMA Psychiatry*, *74*(10), 987–995. https://doi.org/10.1001/jamapsychiatry.2017.2150

DeBar, L. L., Wilson, G. T., Yarborough, B. J., Burns, B., Oyler, B., Hildebrandt, T., Clarke, G. N., Dickerson, J., & Striegel, R. H. (2013). Cognitive Behavioral Treatment for Recurrent Binge Eating in Adolescent Girls: A Pilot Trial. *Cognitive and Behavioral Practice*, *20*(2), 147–161. https://doi.org/10.1016/j.cbpra.2012.04.001

Devlin, M. J., Goldfein, J. A., Petkova, E., Jiang, H., Raizman, P. S., Walk, S., Mayer, L., Carino, J., Bellace, D., Kamenetz, C., Dobrow, I., & Walsh, B. T. (2005). Cognitive behavioral therapy and fluoxetine as adjuncts to group behavioral therapy for binge eating disorder. *Obesity Research*, *13*(6), 1077–1088. https://doi.org/10.1038/oby.2005.126

Dingemans, A. E., Spinhoven, P., & Furth, E. F. Van. (2007). *Predictors and mediators of treatment outcome in patients with binge eating disorder*. *45*, 2551–2562. https://doi.org/10.1016/j.brat.2007.06.003

Eldredge, K. L., Agras, W. S., Telch, C. F., & Marnell, M. (1997). *The Effects of Extending Cognitive-Behavioral Therapy for Binge Eating Disorder Among Initial Treatment Nonresponders*.

Freeman, C. P. L., Barry, F., & Henderson, A. (1988). *Controlled trial of psychotherapy for bulimia nervosa*. *296*(February).

Gorin, A. A., Grange, D. Le, & Stone, A. A. (2002). *Effectiveness of Spouse Involvement in Cognitive Behavioral Therapy for Binge Eating Disorder*. https://doi.org/10.1002/eat.10152

Griffiths, R. A., Hadzispavlovic, D., & Channondlittle, L. (1994). *A Controlled Evaluation of Hypnobehavioural Treatment for Bulimia Nervosa : Immediate Pre-Post Treatment Effects*. *2*(4), 202–220.

Grilo, C. M., Masheb, R. M., & Wilson, G. T. (2005). Efficacy of cognitive behavioral therapy and fluoxetine for the treatment of binge eating disorder: A randomized double-blind placebo-controlled comparison. *Biological Psychiatry*, *57*(3), 301–309. https://doi.org/10.1016/j.biopsych.2004.11.002

Hilbert, A., Petroff, D., Neuhaus, P., & Schmidt, R. (2019). *Letter to the Editor*. 9–11. https://doi.org/10.1159/000503116

Hsu, L. K. G., Rand, W., Sullivan, S., Liu, D. W., Mulliken, B., McDonagh, B., & Kaye, W. H. (2001). Cognitive therapy, nutritional therapy and their combination in the treatment of bulimia nervosa. *Psychological Medicine*, *31*(5), 871–879. https://doi.org/10.1017/S003329170100410X

Kenardy, J., Mensch, M., Bowen, K., Green, B., & Walton, J. (2002). Group therapy for binge eating in Type 2 diabetes: A randomized trial. *Diabetic Medicine*, *19*(3), 234–239. https://doi.org/10.1046/j.1464-5491.2002.00679.x

Kirkley, B. G., Schneider, J. A., Agras, W. S., & Bachman, J. A. (1985). Comparison of Two Group Treatments for Bulimia. *Journal of Consulting and Clinical Psychology*, *53*(1), 43–48. https://doi.org/10.1037/0022-006X.53.1.43

Laessle, R. G., Waadt, S., & Pirke, K. M. (1987). A structured behaviorally oriented group treatment for bulimia nervosa. *Psychotherapy and Psychosomatics*, *48*(1–4), 141–145. https://doi.org/10.1159/000288044

Lee, N. F., & Rush, A. J. (1986). Cognitive-behavioral group therapy for bulimia. *International Journal of Eating Disorders*, *5*(4), 599–615. https://doi.org/https://doi.org/10.1002/1098-108X(198605)5:4<599::AID-EAT2260050402>3.0.CO;2-W

Leitenberg, H., Rosen, J. C., Gross, J., Nudelman, S., & Vara, L. S. (1988). *Exposure Plus Response-Prevention Treatment of Bulimia Nervosa*. *56*(4).

Lewer, M., Kosfelder, J., Michalak, J., Schroeder, D., Nasrawi, N., & Vocks, S. (2017). *Effects of a cognitive-behavioral exposure- based body image therapy for overweight females with binge eating disorder : a pilot study*. 1–12. https://doi.org/10.1186/s40337-017-0174-y

Mathisen, T. F., Bratland-sanda, S., Rosenvinge, J. H., Friborg, O., Pettersen, G., Vrabel, K. A., & Sundgot-borgen, J. (2018). *Treatment effects on compulsive exercise and physical activity in eating disorders*. *1*, 1–9.

Mitchell, J. E., Pyle, R. L., Eckert, E. D., Hatsukami, D., Pomeroy, C., & Zimmerman, R. (1990). A Comparison Study of Antidepressants and Structured Intensive Group Psychotherapy in the Treatment of Bulimia Nervosa. *Archives of General Psychiatry*, *47*(2), 149–157. https://doi.org/10.1001/archpsyc.1990.01810140049008

Peterson, C. B., Mitchell, J. E., Crow, S. J., Crosby, R. D., & Wonderlich, S. A. (2009). The efficacy of self-help group treatment and therapist-led group treatment for  binge eating disorder. *The American Journal of Psychiatry*, *166*(12), 1347–1354. https://doi.org/10.1176/appi.ajp.2009.09030345

Peterson, C. B., Mitchell, J. E., Engbloom, S., Nugent, S., Mussell, M. P., & Miller, J. P. (1997). *Group Cognitive-Behavioral Treatment of Binge Eating Disorder : A Comparison of Therapist-Led Versus Self-Help Formats*.

Schag, K., Rennhak, S. K., Leehr, E. J., Skoda, E. M., Becker, S., Bethge, W., Martus, P., Zipfel, S., & Giel, K. E. (2019). IMPULS: Impulsivity-focused group intervention to reduce binge eating episodes in patients with binge eating disorder-a randomised controlled trial. *Psychotherapy and Psychosomatics*, *88*(3), 141–153. https://doi.org/10.1159/000499696

Schlup, B., Munsch, S., Meyer, A. H., Margraf, J., & Wilhelm, F. H. (2009). Behaviour Research and Therapy The efficacy of a short version of a cognitive-behavioral treatment followed by booster sessions for binge eating disorder. *Behaviour Research and Therapy*, *47*(7), 628–635. https://doi.org/10.1016/j.brat.2009.04.003

Shapiro, J. R., Reba-harrelson, L., Dymek-valentine, M., Woolson, S. L., Hamer, R. M., & Bulik, C. M. (2007). *Feasibility and Acceptability of CD-ROM-Based Cognitive-Behavioural Treatment for Binge-Eating Disorder*. *184*(February), 175–184. https://doi.org/10.1002/erv

Shelley-Ummenhofer, J., & MacMillan, P. D. (2007). Cognitive-behavioural treatment for women who binge eat. *Canadian Journal of Dietetic Practice and Research : A Publication of Dietitians  of Canada = Revue Canadienne de La Pratique et de La Recherche En Dietetique : Une Publication Des Dietetistes Du Canada*, *68*(3), 139–142. https://doi.org/10.3148/68.3.2007.139

Sundgot-Borgen, J., Rosenvinge, J. H., Bahr, R., & Schneider, L. S. (2002). The effect of exercise, cognitive therapy, and nutritional counseling in treating  bulimia nervosa. *Medicine and Science in Sports and Exercise*, *34*(2), 190–195. https://doi.org/10.1097/00005768-200202000-00002

Tasca, G. A., Ritchie, K., Conrad, G., Balfour, L., Lybanon, V., Bissada, H., Tasca, G. A., Ritchie, K., Conrad, G., Balfour, L., Tasca, G. A., Ritchie, K., Conrad, G., Balfour, L., Gayton, J., Lybanon, V., & Bissada, H. (2007). *Attachment scales predict outcome in a randomized controlled trial of two group therapies for binge eating disorder : An aptitude by treatment interaction treatment interaction*. *3307*. https://doi.org/10.1080/10503300500090928

Telch, C. F., Agras, W. S., Rossiter, E. M., Wilfley, D., & Kenardy, J. (1990). Group cognitive-behavioral treatment for the nonpurging bulimic: an initial  evaluation. *Journal of Consulting and Clinical Psychology*, *58*(5), 629–635. https://doi.org/10.1037//0022-006x.58.5.629

Ter Huurne, E. D., De Haan, H. A., Postel, M. G., Van Der Palen, J., Van Der Nagel, J. E. L., & DeJong, C. A. J. (2015). Web-based cognitive behavioral therapy for female patients with eating disorders: Randomized controlled trial. *Journal of Medical Internet Research*, *17*(6), e152. https://doi.org/10.2196/jmir.3946

Thackwray, D. E., Smith, M. C., Bodfishk, J. W., & Meyers, A. W. (1993). A Comparison of Behavioral and Cognitive-Behavioral Interventions for Bulimia Nervosa. *Journal of Consulting and Clinical Psychology*, *61*(4), 639–645. https://doi.org/10.1037/0022-006X.61.4.639

Treasure, J., Schmidt, U., Troop, N., Tiller, J., Todd, G., Keilen, M., & Dodge, E. (1994). First step in managing bulimia nervosa: controlled trial of therapeutic manual. *BMJ (Clinical Research Ed.)*, *308*(6930), 686–689. https://doi.org/10.1136/bmj.308.6930.686

Wagner, B., Nagl, M., Ruth, D., Klinitzke, G., Hilbert, A., & Kersting, A. (2016). NU SC. *Behavior Therapy*. https://doi.org/10.1016/j.beth.2016.01.006

Wilfley, D. E., Agras, W. S., Telch, C. E., Rossiter, E. M., Schneider, J. A., Cole, A. G., Sifford, L., Raeburn, S. D., Wilfley, D. E., Agras, W. S., Telch, C. F., Ros-, E. M., Schneider, J. A., Cole, A. G., & Sifford, L. (1993). *Group Cognitive-Behavioral Therapy and Group Interpersonal Psychotherapy for the Nonpurging Bulimic Individual : A Controlled Comparison*. *6*(2), 296–305.

Wolf, E. M., & Crowther, J. H. (1992). *An Evaluation of Behavioral and Cognitive-Behavioral Group Interventions for the Treatment of Bulimia Nervosa in Women*. *11*(1), 3–15.

Zerwas, S. C., Watson, H. J., Hofmeier, S. M., Levine, M. D., Hamer, R. M., Crosby, R. D., Runfola, C. D., Peat, C. M., Shapiro, J. R., Zimmer, B., Moessner, M., Kordy, H., Marcus, M. D., & Bulik, C. M. (2016). CBT4BN: A Randomized Controlled Trial of Online Chat and Face-to-Face Group Therapy for Bulimia Nervosa. *Psychotherapy and Psychosomatics*, *86*(1), 47–53. https://doi.org/10.1159/000449025

## **SUPPLEMENT 6: Risk of bias assessment in included studies**

**Table S4.** Formula for calculation of Overall Risk of Bias

| Global rating | Items with high risk | Items with unclear risk |
| --- | --- | --- |
| low risk | 0 | =< 3 |
| moderate | 0 | >3 |
| moderate | 1 | any |
| high risk | >1 | any |

**Figure S2**. Risk of bias Summary plot

**Figure S3.1** Risk of bias traffic light plot (I)

**Figure S3.2** Risk of bias traffic light plot (II)

## **SUPPLEMENT 7: Forest plots for all outcomes**

7.1. Primary Outcomes

7.1.1. Remission

**Figure S4**. Forest plot – remission rates, pooled result.

Note. RR = risk ratio with 95% CI (Confidence Intervals).

**Figure S4.1**. Forest plot – resmission rates, subgroup analysis by control condition.

Note. RR = risk ratio with 95% CI (Confidence Intervals).

**Figure S4.2.** Forest plot – remission rates, subgroup analysis per diagnosis.

Note. RR = risk ratio with 95% CI (Confidence Intervals).

**Figure S4.3**. Forest plot – remission rates, subgroup analysis by trial duration.

Note. RR = risk ratio with 95% CI (Confidence Intervals).

**Figure S4.4**. Forest plot – remission rates, subgroup analysis by risk of allegiance bias.

Note. RR = risk ratio with 95% CI (Confidence Intervals).

**Figure S4.5**. Forest plot – remission rates, subgroup analysis by risk of detection bias.

Note. RR = risk ratio with 95% CI (Confidence Intervals).

**Figure S4.6**. Forest plot – remission rates, subgroup analysis by risk of attrition bias.

Note. RR = risk ratio with 95% CI (Confidence Intervals).

**Figure S4.7**. Forest plot – remission rates, subgroup analysis by format of CBT delivery.

Note. RR = risk ratio with 95% CI (Confidence Intervals).

**Figure S4.8**. Forest plot – remission rates, sensitivity analysis, excluding studies with per protocol analysis.

Note. RR = risk ratio with 95% CI (Confidence Intervals).

**Figure S4.9**. Forest plot – remission rates, sensitivity analysis excluding studies with high risk of attrition bias.

Note. RR = risk ratio with 95% CI (Confidence Intervals).

7.1.2. Frequency of binge-eating

**Figure S5**. Forest plot – frequency of binge-eating, pooled result.

Note. SMD = standardized mean difference with 95% CI (Confidence Intervals).

**Figure S5.1**. Forest plot – frequency of binge-eating, subgroup analysis by control condition.

Note. SMD = standardized mean difference with 95% CI (Confidence Intervals).

**Figure S5.2.** Forest plot – frequency of binge-eating, subgroup analysis per diagnosis.

Note. SMD = standardized mean difference with 95% CI (Confidence Intervals).

**Figure S5.3**. Forest plot – frequency of binge-eating, subgroup analysis by trial duration.

Note. SMD = standardized mean difference with 95% CI (Confidence Intervals).

**Figure S5.4**. Forest plot – frequency of binge-eating, subgroup analysis by risk of allegiance bias.

Note. SMD = standardized mean difference with 95% CI (Confidence Intervals).

**Figure S5.5**. Forest plot – frequency of binge-eating, subgroup analysis by risk of detection bias.

Note. SMD = standardized mean difference with 95% CI (Confidence Intervals).

**Figure S5.6**. Forest plot – frequency of binge-eating, subgroup analysis by risk of attrition bias.

Note. SMD = standardized mean difference with 95% CI (Confidence Intervals).

**Figure S5.7**. Forest plot – frequency of binge-eating, subgroup analysis by format of CBT delivery.

Note. SMD = standardized mean difference with 95% CI (Confidence Intervals).

**Figure S5.7**. Forest plot – frequency of binge-eating, sensitivity analysis, excluding studies with per protocol analysis.

Note. SMD = standardized mean difference with 95% CI (Confidence Intervals).

**Figure S5.8.** Forest plot – frequency of binge-eating, sensitivity analysis excluding studies with high risk of attrition bias.

Note. SMD = standardized mean difference with 95% CI (Confidence Intervals).

7.2. Secondary Outcomes

7.2.1. Response

**Figure S6**. Forest plot – response rates, pooled result.

Note. RR = risk ratio with 95% CI (Confidence Intervals).

7.2.2. Eating psychopathology (as measured by validated scales)

**Figure S7**. Forest plot – eating psychopathology as measured by validated scales, pooled result.

Note. SMD = standardized mean difference with 95% CI (Confidence Intervals).

7.2.3. Frequency of purging episodes

**Figure S8**. Forest plot – frequency of purging, pooled result.

Note. SMD = standardized mean difference with 95% CI (Confidence Intervals).

7.2.4. Symptoms of Depression (as measured by validated scales)

**Figure S9**. Forest plot – symptoms of depression as measured by validated scales, pooled result.

Note. SMD = standardized mean difference with 95% CI (Confidence Intervals).

7.2.5. Symptoms of Anxiety (as measured by validated scales)

**Figure S10**. Forest plot – symptoms of anxiety as measured by validated scales, pooled result.

Note. SMD = standardized mean difference with 95% CI (Confidence Intervals).

7.2.6. Self-esteem (as measured by validated scales)

**Figure S11**. Forest plot – self-esteem as measured by validated scales, pooled result.

Note. SMD = standardized mean difference with 95% CI (Confidence Intervals).

7.2.7. Quality of life (as measured by validated scales)

**Figure S12**. Forest plot – quality of life as measured by validated scales, pooled result.

Note. SMD = standardized mean difference with 95% CI (Confidence Intervals).

7.2.8. Body weight (measured in kilograms)

**Figure S13**. Forest plot – body weight measured in kilograms, pooled result.

Note. MD = weighted mean difference with 95% CI (Confidence Intervals).

7.2.9. Body mass index (measured in kg/m^2^)

**Figure S14**. Forest plot – Body mass index measured in kg/m^2^, pooled result.

Note. MD = weighted mean difference with 95% CI (Confidence Intervals).

7.2.10. Number of dropouts

**Figure S15**. Forest plot – number of dropouts, pooled result.

Note. RR = risk ratio with 95% CI (Confidence Intervals).

## **SUPPLEMENT 8: Meta-regression**

**8.1. Meta-regression of Remission on Baseline Binge Frequency**

Mixed-Effects Model (k = 19; tau^2 estimator: REML)

Model Results:

estimate	se	zval	pval	ci.lb	ci.ub

intrcpt	0.5014 0.3604 1.3911 0.1642 -0.2050 1.2078

mod	0.0399 0.0752 0.5313 0.5952 -0.1074 0.1872

Signif. codes: 0 ‘***’ 0.001 ‘**’ 0.01 ‘*’ 0.05 ‘.’ 0.1 ‘ ’ 1

I^2 (residual heterogeneity / unaccounted variability): 56.46%

H^2 (unaccounted variability / sampling variability): 2.30

R^2 (amount of heterogeneity accounted for): 0.00%

**Figure S16.** Bubble plot - Meta-regression of Remission on Baseline Binge Frequency

Note. Bubble size proportional to study weight.

**8.1.1. Meta-regression of Remission on Baseline Binge Frequency in studies with waiting list as control**

Mixed-Effects Model (k = 10; tau^2 estimator: REML)

Model Results:

estimate	se	zval	pval	ci.lb	ci.ub

intrcpt	0.7060 0.4545 1.5534 0.1203 -0.1848 1.5968

mod	0.0960 0.1078 0.8908 0.3731 -0.1153 0.3074

Signif. codes: 0 ‘***’ 0.001 ‘**’ 0.01 ‘*’ 0.05 ‘.’ 0.1 ‘ ’ 1

I^2 (residual heterogeneity / unaccounted variability): 20.49%

H^2 (unaccounted variability / sampling variability): 1.26

R^2 (amount of heterogeneity accounted for): 32.47%

**Figure S16.1.** Bubble plot - Meta-regression of Remission on Baseline Binge Frequency in studies with waiting list as control

Note. Bubble size proportional to study weight.

**8.2. Meta-regression of Remission on Treatment Duration**

Mixed-Effects Model (k = 23; tau^2 estimator: REML)

Model Results:

estimate	se	zval	pval	ci.lb	ci.ub

intrcpt	-0.2669 0.0749 -3.5635 0.0004 -0.4138 -0.1201 ***

mod	0.0541 0.0078	6.9028 <.0001	0.0388	0.0695 ***

Signif. codes: 0 ‘***’ 0.001 ‘**’ 0.01 ‘*’ 0.05 ‘.’ 0.1 ‘ ’ 1

I^2 (residual heterogeneity / unaccounted variability): 0.00%

H^2 (unaccounted variability / sampling variability): 1.00

R^2 (amount of heterogeneity accounted for): 100.00%

**Figure S17.** Bubble plot - Meta-regression of Remission on Treatment duration

Note. Bubble size proportional to study weight.

**8.2.1. Meta-regression of Remission on Treatment Duration in studies with waiting list as control**

Mixed-Effects Model (k = 11; tau^2 estimator: REML)

Model Results:

estimate	se	zval	pval	ci.lb	ci.ub

intrcpt	2.1886 0.8213	2.6648 0.0077	0.5789 3.7984 **

mod	-0.0567 0.0447 -1.2694 0.2043 -0.1444 0.0309

Signif. codes: 0 ‘***’ 0.001 ‘**’ 0.01 ‘*’ 0.05 ‘.’ 0.1 ‘ ’ 1

I^2 (residual heterogeneity / unaccounted variability): 31.56%

H^2 (unaccounted variability / sampling variability): 1.46

R^2 (amount of heterogeneity accounted for): 0.00%

**Figure S17.1.** Bubble plot - Meta-regression of Remission on Baseline Binge Frequency

Note. Bubble size proportional to study weight.

**8.3. Meta-regression of Remission on Baseline BMI**

Mixed-Effects Model (k = 12; tau^2 estimator: REML)

Model Results:

estimate	se	zval	pval	ci.lb	ci.ub

intrcpt	0.3776 1.1886 0.3177 0.7507 -1.9520 2.7073

mod	0.0068 0.0334 0.2034 0.8388 -0.0587 0.0723

Signif. codes: 0 ‘***’ 0.001 ‘**’ 0.01 ‘*’ 0.05 ‘.’ 0.1 ‘ ’ 1

I^2 (residual heterogeneity / unaccounted variability): 66.10%

H^2 (unaccounted variability / sampling variability): 2.95

R^2 (amount of heterogeneity accounted for): 0.00%

**Figure S18.** Bubble plot - Meta-regression of Remission on Baseline BMI

Note. Bubble size proportional to study weight.

**8.4. Meta-regression of Binge Frequency on Baseline Binge Frequency**

Mixed-Effects Model (k = 27; tau^2 estimator: REML)

Model Results:

estimate	se	zval	pval	ci.lb	ci.ub

intrcpt	-0.7556 0.2382 -3.1713 0.0015 -1.2225 -0.2886 **

mod	0.0415 0.0450	0.9219 0.3566 -0.0467	0.1296

Signif. codes: 0 ‘***’ 0.001 ‘**’ 0.01 ‘*’ 0.05 ‘.’ 0.1 ‘ ’ 1

I^2 (residual heterogeneity / unaccounted variability): 69.43%

H^2 (unaccounted variability / sampling variability): 3.27

R^2 (amount of heterogeneity accounted for): 0.00%

**Figure S19.** Bubble plot - Meta-regression of Binge Frequency on Baseline Binge Frequency

Note. Bubble size proportional to study weight.

**8.4.1. Meta-regression of Binge Frequency on Baseline Binge Frequency in studies with waiting list as control**

Mixed-Effects Model (k = 17; tau^2 estimator: REML)

Model Results:

estimate	se	zval	pval	ci.lb	ci.ub

intrcpt	-1.0071 0.1735 -5.8033 <.0001 -1.3472 -0.6669 ***

mod	0.0394 0.0347	1.1339 0.2568 -0.0287	0.1075

Signif. codes: 0 ‘***’ 0.001 ‘**’ 0.01 ‘*’ 0.05 ‘.’ 0.1 ‘ ’ 1

I^2 (residual heterogeneity / unaccounted variability): 0.01%

H^2 (unaccounted variability / sampling variability): 1.00

R^2 (amount of heterogeneity accounted for): 99.82%

**Figure S19.1.** Bubble plot - Meta-regression of Binge Frequency on Baseline Binge Frequency in studies with waiting list as control

Note. Bubble size proportional to study weight.

**8.5. Meta-regression of Binge Frequency on Treatment Duration**

Mixed-Effects Model (k = 33; tau^2 estimator: REML)

Model Results:

estimate	se	zval	pval	ci.lb	ci.ub

intrcpt	-0.6659 0.3199 -2.0812 0.0374 -1.2929 -0.0388 *

mod	0.0055 0.0216	0.2554 0.7984 -0.0368	0.0478

Signif. codes: 0 ‘***’ 0.001 ‘**’ 0.01 ‘*’ 0.05 ‘.’ 0.1 ‘ ’ 1

I^2 (residual heterogeneity / unaccounted variability): 74.30%

H^2 (unaccounted variability / sampling variability): 3.89

R^2 (amount of heterogeneity accounted for): 0.00%

**Figure S20.** Bubble plot - Meta-regression of Binge Frequency on Treatment Duration

Note. Bubble size proportional to study weight.

**8.5.1. Meta-regression of Binge Frequency on Treatment Duration in studies with self-help as a control**

Mixed-Effects Model (k = 10; tau^2 estimator: REML)

Model Results:

estimate	se	zval	pval	ci.lb	ci.ub

intrcpt	0.0314 0.3937	0.0796 0.9365 -0.7402 0.8030

mod	-0.0104 0.0240 -0.4320 0.6657 -0.0573 0.0366

Signif. codes: 0 ‘***’ 0.001 ‘**’ 0.01 ‘*’ 0.05 ‘.’ 0.1 ‘ ’ 1

I^2 (residual heterogeneity / unaccounted variability): 39.24%

H^2 (unaccounted variability / sampling variability): 1.65

R^2 (amount of heterogeneity accounted for): 0.00%

**Figure S20.1.** Bubble plot - Meta-regression of Binge Frequency on Treatment Duration in studies with self-help as a control

Note. Bubble size proportional to study weight.

**8.5.2. Meta-regression of Binge Frequency on Treatment Duration in studies with waiting list as a control**

Mixed-Effects Model (k = 20; tau^2 estimator: REML)

Model Results:

estimate	se	zval	pval	ci.lb	ci.ub

intrcpt	-0.8630 0.3501 -2.4648 0.0137 -1.5492 -0.1768 *

mod	-0.0014 0.0242 -0.0560 0.9553 -0.0488	0.0461

Signif. codes: 0 ‘***’ 0.001 ‘**’ 0.01 ‘*’ 0.05 ‘.’ 0.1 ‘ ’ 1

I^2 (residual heterogeneity / unaccounted variability): 53.81%

H^2 (unaccounted variability / sampling variability): 2.17

R^2 (amount of heterogeneity accounted for): 0.00%

**Figure S20.2.** Bubble plot - Meta-regression of Binge Frequency on Treatment Duration in studies with waiting list as a control

Note. Bubble size proportional to study weight.

**8.6. Meta-regression of Binge Frequency on Baseline BMI**

Mixed-Effects Model (k = 16; tau^2 estimator: REML)

Model Results:

estimate	se	zval	pval	ci.lb	ci.ub

intrcpt	0.6007 0.6741	0.8911 0.3729 -0.7205 1.9218

mod	-0.0340 0.0193 -1.7657 0.0774 -0.0718 0.0037 .

Signif. codes: 0 ‘***’ 0.001 ‘**’ 0.01 ‘*’ 0.05 ‘.’ 0.1 ‘ ’ 1

I^2 (residual heterogeneity / unaccounted variability): 75.10%

H^2 (unaccounted variability / sampling variability): 4.02

R^2 (amount of heterogeneity accounted for): 20.55%

**Figure S20.2.** Bubble plot - Meta-regression of Binge Frequency on Treatment

Note. Bubble size proportional to study weight.

## **SUPPLEMENT 9: Publication bias**

9.1. Remission rates

**Figure S22.1** Funnel plot – remission rates

**Figure S22.2** Trim-and-fill adjusted Funnel plot – remission rates

**Figure S22.3** Egger’s regression plot – remission rates

Egger’s test – remission rates

Model: weighted regression with multiplicative dispersion

Predictor: standard error

Test for Funnel Plot Asymmetry: t = 6.9049, df = 21, p < .0001

Limit Estimate (as sei -> 0): b = -0.0684 (CI: -0.1793, 0.0425)

9.2. Frequency of binge-eating

**Figure S23.1** Funnel plot – frequency of binge-eating

**Figure S23.2** Trim-and-fill adjusted Funnel plot – frequency of binge-eating

**Figure S23.3** Egger’s regression plot – frequency of binge-eating

Egger’s test – frequency of binge-eating

Model: weighted regression with multiplicative dispersion

Predictor: standard error

Test for Funnel Plot Asymmetry: t = -1.5307, df = 31, p = 0.1360

Limit Estimate (as sei -> 0): b = -0.1750 (CI: -0.6737, 0.3237)

## **SUPPLEMENT 10: GRADE**

**Table S5.** Summary of findings
